# A single-entry model and wait time for hip and knee replacement in eastern health region of Newfoundland and Labrador 2011–2019

**DOI:** 10.1186/s12913-021-07451-8

**Published:** 2022-01-16

**Authors:** Anh Thu Vo, Yanqing Yi, Maria Mathews, James Valcour, Michelle Alexander, Marcel Billard

**Affiliations:** 1 Faculty of Medicine, Memorial University of Newfoundland, St. John’s, Canada; 2 Schulich School of Medicine & Dentistry, Western University, London, Canada; 3 Clinical Efficiency Program, Eastern Health, London, Canada; 4 Central Intake Division, Clinical Efficiency Program, Eastern Health, London, Canada

**Keywords:** Single-entry model, Priority levels, Wait time, Consultation, Surgery, Total knee replacement, Total hip replacement, Survival analysis

## Abstract

**Background:**

A single-entry model in healthcare consolidates waiting lists through a central intake and allows patients to see the next available health care provider based on the prioritization. This study aimed to examine whether and to what extent the prioritization reduced wait times for hip and knee replacement surgeries.

**Method:**

The survival regression method was used to estimate the effects of priority levels on wait times for consultation and surgery for hip and knee replacements. The sample data included patients who were referred to the Orthopedic Central Intake clinic at the Eastern Health region of Newfoundland and Labrador and had surgery of hip and knee replacements between 2011 and 2019.

**Result:**

After adjusting for covariates, the hazard of having consultation booked was greater in patients with priority 1 and 2 than those in priority 3 when and at 90 days after the referral was made for both hip and knee replacements. Regarding wait time for surgery after the decision for surgery was made, while the hazard of having surgery was lower in priority 2 than in priority 3 when and indifferent at 182 days after the decision was made, it was not significantly different between priority 1 and priority 3 among hip replacement patients. Priority levels were not significantly related to the hazard of having surgery for a knee replacement after the decision for surgery was made. Overall, the hazard of having surgery after the referral was made by a primary care physician was greater for patients in high priority than those in low priority. Preferring a specific surgeon indicated at referral was found to delay consultation and it was not significantly related to the total wait time for surgery. Incomplete referral forms prolonged wait time for consultation and patients under age 65 had a longer total wait time than those aged 65 or above.

**Conclusion:**

Patients with high priority could have a consultation booked earlier than those with low priority and prioritization in a single entrance model shortens the total wait time for surgery. However, the association between priority levels and wait for surgery after the decision for surgery was made has not well-established.

## Background

Canada’s publicly funded healthcare system ensures that all eligible residents in provinces and territories have access to medically necessary hospital and physician services without financial barriers. However, Canada has the longest wait times for specialists and non-emergency surgeries compared to other OECD countries (Organization for Economic Cooperation and Development) [1]. Over a half of patients had to wait over 9 months for their hip and knee replacement (HKR) surgeries [2–15].

Long wait times for HKR surgery might be associated with an increase in medical and non-medical costs and a deterioration in health-related quality of life. A total cost of medications for patients with long wait times is higher than those with short wait times [16, 17]. Approximately 20% of patients in the workforce waiting for a hip replacement surgery have to be off of work because of negative health consequences [16]. Patients with longer wait times for HKR have difficulties with mobility, pain while waiting for their surgery [16, 18, 19].

During the past few decades, the Canadian government has made considerable effort to improve wait times for HKR. The Health Ministers announced evidence-based national benchmark of 182 days for HKR surgery [20, 21]. The National Standards Committee of the Canadian Orthopedic Association (COA) also recommended benchmarks for maximum acceptable wait time (MAWT), including two intervals: an MAWT benchmark within 90 days for consultation, and an MAWT benchmark within 182 days for surgery [22]. Most provinces have developed strategies to strive to meet the benchmarks. A central intake system, known as a single-entry model (SEM), is an innovation for managing wait times [23]. Under traditional models, surgeons receive referrals and manage their own waiting list. When a patient is referred to a surgeon whose appointment slots are all unavailable, the patient has to wait until a vacancy is available, even if another surgeon is available [24]. In contrast, in the SEM, multiple queues are consolidated into a single queue through a central intake system, and patients can meet the next available orthopedic surgeon based on their urgent conditions [25–27]. Many studies show that the SEM improved wait time for consultation (WT1) [28–31], wait time for surgery (WT2) [27–32], and a total wait time (TW) for HKR through the SEM [27, 29]. Wait time for consultation (WT1) is the duration from the date when the surgeon’s office, or central intake receives the referral [33–37], or when the referral is made by family doctor [38, 39] to the date when a patient has consultation with surgeon [33–39]. Wait time for surgery is defined as a wait time period from the date when a surgeon and a patient decide to surgery [33–38], or when a booking form is received by the health authority [39] to the date when a patient receives surgery [33–39].

The Eastern Health region of Newfoundland and Labrador implemented an Orthopedic Central Intake (OCI) clinic and developed a routine priority classification to triage patients to a surgeon for consultation based on the patient’s urgency level since 2011. An interdisciplinary assessment team in the OCI clinic, including a nurse, social worker, physiotherapist, occupational therapist and appointment clerk, assessed the urgency for surgery considering stage pathology or complex MSK issue, level of dysfunction, and whether conservative treatment opinions failed based on the information on the referral form from the primary physicians and the results of X-ray if possible as well as other information collected during the team’s interaction with patients after a referral was made. Then the team assigned patients with highest priority routine referral as Priority 1 (end stage pathology or complex MSK issue, high level of dysfunction, and conservative treatment opinions failed); those with moderate priority routine referral as priority 2 (moderate to end stage pathology or complex MSK issue, moderate to high functional impairment despite best conservative); those with low priority routine referral as priority 3 (early to moderate stage pathology, moderate functional impairment, minimal evidence of conservative management trialed or currently managing with conservative interventions); and priority 4 for not appropriate for orthopedic surgical consultation (early-stage disease, minimal symptoms/functional impairment, minimal evidence of conservative intervention trialed and/or patient responding to conservative treatment). According to the priority classification, patients with priority 1, 2, and 3 would be booked for an appointment with a surgeon within 45 days, 90 days, and 6 months to under 12 months of receipt of the complete referral, respectively. Patients with priority 4 would not be considered suitable for consultation at triage assessment, but they could be booked an appointment for consultation if they do not respond to their non-surgery treatment [40]. Based on the annual reports of Eastern Health (2011–2014) [41–43], the median WT1 decreased from 95 days to 47 days for priority 1 and 2, and from 182 days to 123 days for priority 3 and 4.

There are challenges in using priority tools for managing wait times. First, patients with low priority might never reach the top of the waiting list when surgeons see a high volume of patients with high priority [44]. Cipriano et al. [45] demonstrated that after 5 year of implementing strict clinical prioritization, the number of patients receiving surgery within MAWT increased in patients with higher urgent scores, but decreased in patients with lower urgent scores. However, the OCI clinic at Eastern Health has not tracked WT2 by priority and, as a result, little is known about whether patients assigned a higher priority level receive surgeries sooner than those with a lower priority level. Second, most studies used descriptive analyses to examine WT1 [28–31], WT2 [29–31, 46], and TW [27, 29]. Consequently, influential factors have not been controlled in these studies [28–32, 46]. One study used a linear regression model to estimate WT2 controlling explanatory variables, but not WT1 and TW [27]. This study addressed these gaps in literature by using a survival analysis to examine whether and to what extent the priority classification in the single-entry model reduced wait times for hip and knee replacements while adjusting for covariates of age, diagnosis type, patient’s preference, initial referral form status, and year of referral.

## Method

### Design and data sources

A secondary data analysis was conducted based on a linking data from the Orthopedic Central Intake (OCI) database and the Total Joint Assessment Center (TJAC) database for referrals sent to the OCI clinic in Eastern Health Region from 2011 to 2019. Using Medical Care Plan (MCP) numbers, or hospitalization number in the event of MCP numbers missing, the OCI team linked the OCI database with the TJAC database by type of surgery (hip or knee). Next, they assigned study identification numbers to patients in the linked cohort and removed MCP numbers and patient’s names. They provided the research team a de-identified database containing variables for data analysis purposes (Table 1).

**Table 1:** Variables for data analysis

Data sources	Variables	Coding	Describe
The OCI database	Primary affected joints	Hip = 1 Knee = 0^a^	Information was *in the Orthopedic Central Intake Patient Referral form.*
	Diagnosis	Osteoarthritis = 1 Others = 0^a^	Information was in *the Orthopedic Central Intake Patient Referral form.*
	Age	< 65 = 1 ≥ 65 = 0^a^	We chose the age threshold at 65 for two reasons. First, from a methodological viewpoint, one of the most practical ways of defining a senior is the age marker of 65. Second, from a conceptual viewpoint, defining the individuals aged 65 receive full pension benefits in Canada [47].
	Patient’s preference	Next available = 1 Specific surgeon = 0^a^	*The SEM provides patients with two choices: the ‘next available surgeon’ and ‘a specific surgeon’ on the Orthopedic Central Intake Patient Referral form.*
	*The date on the referral form*		*WT1 starts when a family doctor refers a patient to the OCI clinic. To measure the starting point of WT1, the referral date to the OCI clinic was used.*
	The date of the first consultation		The date of the first consultation with an orthopedic surgeon was used to measure an ending point of WT1.
	Initial referral form	Incomplete = 1 Complete = 0^a^	Incomplete referral form status was indicated by incomplete clerical dates. Based on this information, it is possible to know whether a referral form was or was not completed
	Priority	P1 = 1 P2 = 2 P4 = 3 P3 = 0^a^	In the Eastern Health region, the routine priority classification has four categories: priority 1 – the highest priority; priority 2 – moderate priority; priority 3 – low priority; and priority 4 – probably unsuitable for surgery
	Year of referral	2011–2013 = 1 2014–2016 = 2 2017–2019 = 0^a^	The year when patients were referred by family doctors, as indicated in *the Orthopedic Central Intake Patient Referral form*
The TJAC database	Date of a decision to treat		WT2 begins on the date when a surgeon and a patient decide to have surgery.
	Date of surgery		The date of surgery was used to measure the endpoint of WT2.

Note. ^a^ Reference group

### Inclusion/exclusion criteria

A cohort of adults aged 18 and older were chosen, all of whom were referred to the OCI Clinic at Eastern Health for HKR assessment between 2011 and 2019. Patients were excluded if they had primary problems with other joints (shoulder, neck, and ankle), or if they were referred for other reasons, including those obtaining partial hip or knee replacements and/or revisions, or those who had urgent referrals booked directly through the hospital.

### Sample size

**Fig. 1:**
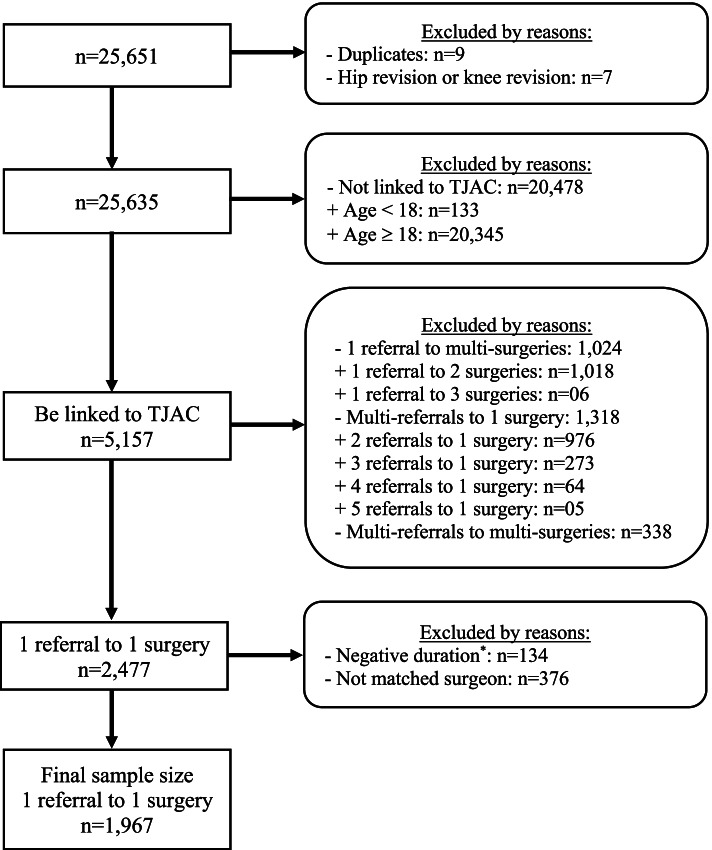
Derivation of the study sample. *duration is a length of time from the date of the first consultation to the date of decision to treat

A total of 1967 referrals that were linked to 1967 individual surgeries were included in the analysis. We excluded a number of referrals because of redundancy (see Fig. 1), including one referral linked to multiple surgeries; multiple referrals linked to one surgery; and multiple referrals linked to multiple surgeries because we could not know which pair of referrals and surgeries was a true match.

### Outcomes

We measured WT1 as the time between the date of the first consultation and the date of referral made by physician; WT2 as the time from the date of decision to surgery to the date of surgery; and a TW as the time between the date of referral and the date of surgery.

### Statistical analysis

First, a description of HKR participants were conducted using frequencies and percentages for categorical variables (age group, diagnosis, initial referral form status, patient’s preference, priority, and year of referrals), means and medians for continuous variables (WT1, WT2, and TW) by categorical groups. The mean wait times were based on Product-limit estimates. We also reported median wait times because the distribution of wait time data tends to be positively skewed, which means may be affected by a few cases waiting a long time [48, 49]. Second, regression models for wait times were used to study factors associated with wait time. The Cox regression models for WT1 and TW contained variables: age group, diagnosis, patient’s preference, initial referral form status, year of referral, and priority. Initial referral form status was assumed to not impact WT2. In fact, the initial referral form status was assessed by the OCI clinic for triaging patients and would be re-sent to physicians if it was not completed, which might prolong WT1, and probably increase a TW. We assessed the proportional hazard assumption of Cox proportional hazard model. With any covariate violating the assumption, we added their interaction with time to the model. This method of adding the model interaction of covariates with time is considered as a potential way to examine the proportional hazard assumption and to address non-proportional issues [50].

## Results

### Patients’ characteristics

Table 2 presents characteristics of patients and the descriptive statistics for wait times of the total hip replacement surgery group (*n* = 808) and the total knee replacement surgery group (*n* = 1159) between 2011 and 2019 in the OCI clinic. In the hip replacement group, patients aged 65 or older accounted for 55.07% (*n* = 445), and hip osteoarthritis accounted for 88.61% (*n* = 716). When assigned priority for consultation, 66.46% (*n* = 537) of patients received priority 2, 24.13% (*n* = 195) of patients received a priority 1, 9.16% (*n* = 74) of patients received priority 3, and 0.25% (*n* = 2) of patients were assigned a priority 4. Only 3.96% (*n* = 32) of patients had incomplete referral form, and 72.03% (*n* = 582) were referred to the next available surgeon for a consultation. 29.58% (*n* = 239) of referrals to the OCI clinic were sent in 2011–2013, 37.87% (*n* = 306) in 2014–2016, and 32.55% (*n* = 263) in 2017–2019.

**Table 2:** A summary for participants with hip and knee replacement surgery

Variables	n (%)	Wait time for consultation			Wait time for surgery			Total wait time		
		Mean (SD)^**a**^	Median^**a**^ (Q1-Q3)	p^**b**^	Mean (SD)^**a**^	Median^**a**^ (Q1-Q3)	p^**b**^	Mean (SD)^**a**^	Median^**a**^ (Q1-Q3)	p^**b**^
**Hip replacement surgery (n = 808)**										
**Age group**				0.0121			0.4088			0.0196
< 65	363 (44.93%)	90.18 (64.43)	77 (49–105)		190.65 (125.70)	164 (101–250)		435.59 (354.16)	327 (215–529)	
≥ 65	445 (55.07%)	80.15 (55.08)	68 (48–97)		183.27 (119.69)	162 (94–246)		381.22 (324.15)	303 (195–458)	
**Diagnosis**				0.3969			0.0073			0.1382
Osteoarthritis	716 (88.61%)	84.14 (57.59)	73 (49–99)		190.53 (124.03)	164 (100–254)		412 (342.68)	317 (209–482)	
Others	92 (11.39%)	88.66 (73.88)	68.50 (42–115)		155.90 (104.52)	144 (78–205.5)		356.17 (304.38)	269 (180–456)	
**Priority**				<.0001*			0.0206*			<.0001*
P1	195 (24.13%)	57.22 (36.45)	49 (32–78)		180.02 (140.78)	148 (89–232)		370.84 (376.59)	269 (155–444)	
P2	537 (66.46%)	77.19 (32.30)	75 (53–97)		192.88 (113.54)	176 (104–260)		391.28 (302.70)	315 (218–463)	
P3	74 (9.16%)	203.89 (94.87)	194.5 (139–267)		155.54 (128.27)	133 (93–177)		582.91 (411.57)	476 (279–739)	
P4	2 (0.25%)	352.5 (154.86)	352.5 (243–462)		283 (127.28)	283 (193–373)		1097.5 (166.17)	1097.5 (980–1215)	
**Initial referral**^**c**^				0.0430			0.5594			0.2996
Incomplete	32 (3.96%)	104.06 (45.49)	99 (67–133.5)		177.03 (92.77)	176 (90.5–227.5)		343.75 (177.65)	321 (197–433.5)	
Complete	776 (96.04%)	83.86 (60.04)	71.5 (49–98)		186.98 (123.51)	162.5 (99–249.5)		408.20 (343.68)	313.5 (205.5–483)	
**Patient’s preference**				0.5281			0.0296			0.3744
Next available	582 (72.03%)	84.15 (59.92)	73 (49–98)		193.12 (121.89)	169 (104–260)		411.24 (335.57)	316 (211–486)	
Specific surgeon	226 (27.97%)	85.96 (59.01)	74.5 (49–105)		169.76 (122.39)	149 (80–225)		391.24 (342.38)	292.5 (178–462)	
**Year of referral**^**d**^				<.0001			<.0001			<.0001
2011–2013	239 (29.58%)	114.05 (65.43)	97 (73–139)		169.03 (131.06)	139 (92–207)		518.70 (457.73)	349 (223–621)	
2014–2016	306 (37.87%)	79.25 (60.92)	61.5 (47–96)		217.21 (125.84)	199.5 (120–288)		431.72 (309.50)	359 (243–504)	
2017–2019	263 (32.55%)	64.24 (38.78)	58 (41–85)		166.90 (101.72)	152 (87–227)		272.57 (146.85)	246 (162–349)	
**Knee replacement surgery (n = 1159)**										
**Age group**				0.0003			0.1092			<.0001
< 65	560 (48.32%)	122.59 (82.27)	97 (68–148)		227.25 (136.47)	209 (123–297)		667 (458.13)	541 (339–885)	
≥ 65	599 (51.68%)	105.78 (71.85)	89 (58–117)		215.79 (127.61)	197 (125–288)		532.30 (395)	412 (271–646)	
**Diagnosis**				0.0219			0.2926			<.0001
Osteoarthritis	1027 (88.61%)	111.85 (75.84)	92 (61–125)		222.93 (131.82)	206.00 (125–296)		577.41 (417.56)	452 (296–730)	
Others	132 (11.39%)	129.84 (87.96)	109 (66.5–165)		208.89 (133.50)	183 (101.5–275.5)		753.42 (508.36)	704 (323–1015)	
**Priority**				<.0001*			0.4233*			<.0001*
P1	116 (10.01%)	68.47 (43.10)	54 (37.5–96)		204.30 (146.90)	178 (113–479)		454.77 (365.14)	324.5 (208–604.5)	
P2	754 (65.06%)	82.69 (31.45)	82 (58–99)		224.88 (127.01)	214.5 (127–302)		530.07 (393.68)	413 (272–649)	
P3	283 (24.42%)	210.64 (83.50)	202 (145–268)		218.60 (138.38)	196 (120–288)		824.47 (463.23)	719 (492–1021)	
P4	6 (0.52%)	352.33 (114.00)	382.5 (355–393)		233 (148.75)	231.5 (106–297)		1117.17 (604.49)	853 (699–1631)	
**Initial referral**^**c**^				0.0686			0.2427			0.0446
Incomplete	68 (5.87%)	132.74 (68.98)	114.5 (78.5–172)		243.99 (144.89)	229.5 (177.5–304.5)		718 (498.09)	550.5 (341–1050)	
Complete	1091 (94.13%)	112.73 (77.86)	92 (60–126)		219.92 (131.13)	201 (122–290)		589.94 (426.95)	464 (296–771)	
**Patient’s preference**				0.2654			0.0030			0.0748
Next available	776 (66.95%)	108.99 (73.63)	95 (61–123)		205.85 (126.07)	183 (116–267)		612.71 (446.54)	484.5 (306–790.5)	
Specific surgeon	383 (33.05%)	116.32 (79.26)	92 (61.5–136.5)		228.97 (134.30)	217 (127–304)		566.56 (400.56)	443 (278–755)	
**Year of referral**^**d**^				<.0001			<.0001			<.0001
2011–2013	396 (34.17%)	144.87 (81.52)	126 (84.5–198.5)		210.47 (149.60)	180.5 (108.5–273.5)		765.59 (563.02)	604 (339–1045.5)	
2014–2016	473 (40.81%)	99.97 (69.18)	84 (55–110)		251.27 (127.57)	246 (155–328)		610.43 (334.31)	532 (364–804)	
2017–2019	290 (25.02%)	94.34 (71.73)	82 (57–98)		187.33 (99.47)	176 (108–249)		346.71 (175.47)	329.5 (207–437)	

^a^Estimated mean and median wait times from Kaplan-Meier analysis

^b^*p*-value for differences in survival functions by categorical groups from log-rank tests *p-value for differences in survival functions among three groups: P1-P3 from the log-rank tests

^c^A standard referral form status when it was sent to the OCI at the first time by family doctors

^d^The year when a patient was referred to the OCI for a hip or knee replacement surgery assessment

In the knee replacement group, 51.68% (*n* = 599) was 65 years or older, and knee osteoarthritis made up 88.61% (*n* = 1027). 65.06% (*n* = 754) of patients were assigned a priority 2, while 24.42% (*n* = 283) of patients were assigned a priority 3, 10.01% (*n* = 116) received a priority 1, 0.52% (*n* = 6) received a priority 4. Incomplete initial referral form made up only 5.87% (*n* = 68), and the percentage of patients choosing the next available surgeon was 66.95% (*n* = 776). Referrals sent to the OCI clinic in 2014–2016 accounted for 40.81% (*n* = 473), 34.17% (*n* = 396) in 2011–2013, and 25.02% (*n* = 290) in 2017–2019.

Priority 4 (n = 2) for hip replacement and (n = 6) for knee replacement were excluded in the survival analysis because of the small sample sizes.

A priority tool for referral allowed patients with higher urgent level to meet a surgery for consultation sooner than their counterparts. In hip replacement group, the shortest median WT1 was 49 days in patients with priority 1, followed by 75 days in patients with priority 2, and 194.5 days in patients with priority 3. For knee replacement group, patients with priority 1 had the shortest WT1 at 54 days, followed by 82 days in patients with priority 2, and 202 days in patients with priority 3 (Table 2). In hip replacement group, patients with priority 2 had the longest median WT2 at 176 days, followed by patients with priority 1 at 148 days, and priority 3 at 133 days. In contrast, there was no difference in survival functions of WT2 across priority levels in the knee replacement group (*p* = 0.4233) (Table 2).

Along with improving timely access to consultation by priority level, patients with higher priority level at triage assessment had a shorter TW than those with lower levels. The shortest median TW was 269 days in patients with priority 1, while a longer median TW was 315 days in patients with priority 2, and 476 days in patients with priority 3 in hip replacement group. Similarly, the median TW was the shortest at 324.5 days in patients with priority 1, followed by at 413 days in patients with priority 2, and at 719 days in patients with priority 3 in knee replacement group (Table 2).

### Regression analysis

Factors associated with wait times from the regression models were summarized in Tables 3, 4 and 5 for WT1, WT2 and WT, respectively. To simplify notation, CI stands for a 95% confidence interval in the following.

**Table 3:** Factors associated with wait time for consultation in hip and knee replacement surgery

Variables	Hip replacement surgery (*n* = 806)						Knee replacement surgery (*n* = 1153)					
	Unadjusted Hazard Ratios			Adjusted Hazard Ratios^**a**^			Unadjusted Hazard Ratios			Adjusted Hazard Ratios^**a**^		
	HR	95% CI	p	HR	95% CI	p	HR	95% CI	p	HR	95% CI	p
Age group*WT1									0.0344			
*WT1 = 0 day*			0.0086*			0.8034*						0.9088*
< 65	0.830	0.722–0.954		1.018	0.883–1.175		0.682	0.551–0.843		1.007	0.895–1.133	
≥ 65												
*WT1 = 90 days*												
< 65							0.796	0.705–0.899				
≥ 65												
Diagnosis			0.3204			0.2159			0.0484			0.3707
Osteoarthritis	1.117	0.898–1.389		0.869	0.695–1.086		1.202	1.001–1.444		0.917	0.759–1.108	
Others												
Patient’s preference			0.4133			0.0015			0.2651			<.0001
Next available	1.066	0.914–1.244		1.296	1.104–1.522		0.932	0.824–1.055		1.461	1.284–1.662	
Specific surgeon												
Initial referral form*WT1			0.0002						0.0002			0.0080
*WT1 = 0 day*						0.0384**						
Incomplete	0.125	0.043–0.363		0.676	0.467–0.979		0.363	0.217–0.608		0.415	0.237–0.727	
Complete												
*WT1 = 90 days*												
Incomplete	0.673	0.460–0.984					0.652	0.491–0.866		0.659	0.488–0.890	
Complete												
Year of referral^b^*WT1			<.0001			<.0001			<.0001			<.0001
*WT1 = 0 day*												
2011–2013	0.184	0.127–0.268		0.137	0.093–0.202		0.212	0.159–0.283		0.257	0.189–0.351	
2014–2016	0.762	0.554–1.047		0.977	0.711–1.343		0.754	0.582–0.977		0.885	0.684–1.145	
2017–2019												
*WT1 = 90 days*												
2011–2013	0.384	0.314–0.471		0.284	0.227–0.355		0.422	0.356–0.500		0.450	0.372–0.544	
2014–2016	0.705	0.585–0.851		0.830	0.686–1.004		0.878	0.758–1.018		0.942	0.812–1.094	
2017–2019												
Priority*WT1			<.0001			<.0001			<.0001			<.0001
*WT1 = 0 day*												
P1	109.700	37.250–323.063		165.240	53.749–507.997		288.827	119.308–699.210		451.547	184.066–1107.723	
P2	20.253	7.354–55.776		15.746	5.492–45.147		47.870	22.666–101.100		36.533	16.978–78.613	
P3												
*WT1 = 90 days*												
P1	11.696	7.101–19.263		21.831	12.934–36.848		14.782	10.272–21.270		24.428	16.722–35.684	
P2	11.165	7.012–17.780		10.764	6.619–17.504		15.596	11.519–21.116		15.635	11.421–21.404	
P3												

^a^ Extended Cox proportional hazard model adjusted for age group, diagnosis, patient’s preference, initial referral form, year of referral, priority, and their interaction with time, where applicable; ^b^ The year when a patient was referred to the OCI for a hip or knee replacement surgery assessment

* *p*-value for age group

** *p*-value for initial referral form

**Table 4:** Factors associated with wait time for surgery in hip and knee replacement surgery

Variables	Hip replacement surgery (n = 806)						Knee replacement surgery (n = 1153)					
	Unadjusted Hazard Ratios			Adjusted Hazard Ratios^**a**^			Unadjusted Hazard Ratios			Adjusted Hazard Ratios^**a**^		
	HR	95% CI	p	HR	95% CI	p	HR	95% CI	p	HR	95% CI	p
Age group			0.4419			0.2145			0.1278			0.2428
< 65	0.947	0.824–1.088		0.913	0.792–1.054		0.914	0.814–1.026		0.932	0.827–1.049	
≥ 65												
Diagnosis			0.0079			0.0163			0.2560			0.1482
Osteoarthritis	0.745	0.599–0.926		0.759	0.606–0.950		0.899	0.749–1.080		0.870	0.720–1.051	
Others												
Patient’s preference*WT2			0.0316									
*WT2 = 0 day*						0.0045*			0.0024*			0.0229*
Next available	0.645	0.485–0.858		0.798	0.683–0.932		0.826	0.730–0.934		0.863	0.761–0.980	
Specific surgeon												
*WT2 = 182 days*												
Next available	0.848	0.726–0.990										
Specific surgeon												
Year of referral^b^*WT2			0.0001			0.0075			<.0001			<.0001
*WT2 = 0 day*												
2011–2013	1.442	1.028–2.023		1.245	0.875–1.771		1.229	0.903–1.674		1.191	0.870–1.631	
2014–2016	0.544	0.383–0.773		0.540	0.379–0.770		0.482	0.350–0.666		0.487	0.352–0.674	
2017–2019												
*WT2 = 182 days*												
2011–2013	0.968	0.807–1.161		0.948	0.785–1.144		0.812	0.696–0.947		0.780	0.660–0.923	
2014–2016	0.651	0.550–0.771		0.645	0.543–0.765		0.553	0.474–0.646		0.566	0.484–0.662	
2017–2019												
Priority*WT2			<.0001			0.0015			0.0102			
*WT2 = 0 day*												0.3512**
P1	0.685	0.448–1.049		0.676	0.438–1.042		1.337	0.905–1.975		1.012	0.812–1.262	
P2	0.388	0.260–0.579		0.447	0.294–0.679		0.767	0.587–1.002		0.909	0.780–1.058	
P3												
*WT2 = 182 days*												
P1	0.824	0.623–1.089		0.827	0.621–1.102		1.140	0.914–1.420				
P2	0.758	0.538–0.981		0.810	0.619–1.059		0.934	0.811–1.077				
P3												

^a^Extended Cox proportional hazard model adjusted for age group, diagnosis, patient’s preference, year of referral, priority, and their interaction with time, where applicable

^b^The year when a patient was referred to the OCI for a hip or knee replacement surgery assessment

* *p*-value for patient’s preference

** *p*-value for priority

**Table 5:** Factors associated with total wait time in hip and knee replacement surgery

Variables	Hip replacement surgery (n = 806)						Knee replacement surgery (n = 1153)					
	Unadjusted Hazard Ratios			Adjusted Hazard Ratios^**a**^			Unadjusted Hazard Ratios			Adjusted Hazard Ratios^**a**^		
	HR	95% CI	p	HR	95% CI	p	HR	95% CI	p	HR	95% CI	p
Age group*TW									0.0227			
*TW = 0 day*			0.0190*			0.0079*						0.0011*
< 65	0.846	0.736–0.973		0.822	0.712–0.950		0.604	0.492–0.741		0.819	0.726–0.923	
≥ 65												
*TW = 182 days*												
< 65							0.661	0.570–0.767				
≥ 65												
Diagnosis			0.1557			0.0097			0.0002			0.1505
Osteoarthritis	0.854	0.687–1.062		0.740	0.589–0.930		1.416	1.178–1.701		1.150	0.951–1.390	
Others												
Patient’s preference			0.4225			0.4708			0.0695			0.2613
Next available	0.939	0.805–1.095		0.944	0.808–1.103		0.892	0.789–1.009		1.075	0.948–1.220	
Specific surgeon												
Initial referral form			0.3130			0.0606			0.0390			0.6993
Incomplete	1.200	0.842–1.711		1.417	0.985–2.038		0.772	0.604–0.987		0.951	0.739–1.225	
Complete												
Year of referral^b^*TW									0.0002			<.0001
*TW = 0 day*			<.0001**			<.0001**						
2011–2013	0.410	0.340–0.495		0.412	0.340–0.500		0.416	0.288–0.600		0.582	0.398–0.850	
2014–2016	0.502	0.424–0.595		0.519	0.437–0.616		0.336	0.235–0.480		0.331	0.230–0.475	
2017–2019												
*TW = 182 days*												
2011–2013							0.326	0.267–0.397		0.421	0.342–0.519	
2014–2016							0.341	0.284–0.411		0.351	0.291–0.424	
2017–2019												
Priority*TW			0.0010			0.0023			<.0001			<.0001
*TW = 0 day*												
P1	3.412	2.163–5.382		2.999	1.889–4.763		5.628	3.696–8.570		5.166	3.364–7.934	
P2	2.239	1.484–3.377		1.793	1.179–2.728		3.539	2.707–4.625		3.221	2.416–4.294	
P3												
*TW = 272 days*												
P1	2.315	1.684–3.181		2.112	1.524–2.927		3.749	2.824–4.978		3.423	2.566–4.565	
P2	1.918	1.428–2.577		1.615	1.194–2.185		2.723	2.230–3.324		2.298	1.860–2.839	
P3												

^a^Extended Cox proportional hazard model adjusted for age group, diagnosis, patient’s preference, initial referral form, year of referral, priority, and their interaction with time, where applicable

^b^The year when a patient was referred to the OCI for a hip or knee replacement surgery assessment

* *p*-value for age group

** *p*-value for year of referral

Table 3 shows that patients in priority levels 1 and 2 were more likely to have consultation booked earlier than those in priority 3 for both of hip and knee replacements. The hazards ratio (HR) of having consultation significantly changed over time. For hip replacement, after adjusting for age group, diagnosis, patient’s preference, initial referral form status, and year of referral, the hazard of having consultation in patients with priority 1 (HR = 165.240, CI: 53.749–507.997) and priority 2 (HR =15.746, CI: 5.492–45.147) were greater than that in patients with priority 3, initially, and remained significantly greater at 90 days after the referral was make for both priority 1 (HR = 21.831, CI: 12.934–36.848) and priority 2 (HR = 10.764, CI: 6.619–17.504). Similar results were observed for wait time for consultation among patients requesting knee replacement. The HR of having consultation were 451.547 (CI: 184.066–1107.723) and 36.533 (CI: 16.978–78.613) initially for priority 1 and 2 patients, respectively when compared with priority 3 patients, and it decreased to 24.428 (CI: 16.722–35.684) and 15.635 (CI: 11.421–21.404) at 90 days after the referral was made.

For both of hip and knee replacements, age group and diagnosis were not related to WT1 after adjusting for other factors in the model, but association were observed for patient’s preference, initial referral form status, and year of referral groups (Table 3). Patients choosing the next available surgeon were found to have greater hazard of having a consultation than those requesting a specific surgeon (adjusted HR = 1.296, CI: 1.104–1.522 for hip; HR = 1.461, CI: 1.284–1.662 for knee). Incomplete initial referral forms delayed consultation for hip replacement patients (adjusted HR = 0.676, CI: 0.467–0.979). The delay was observed as well for knee replacement consultation initially (adjusted HR = 0.415, CI: 0.237–0.727), and at 90 days after the referral was made (adjusted HR = 0.659, CI: 0.488–0.890). The HR for Year of Referral groups significantly changed over time for both of hip and knee replacements. Referrals made in years of 2011 to 2013 were less likely to have consultation book earlier in comparison with those referred in years of 2017 to 2019 and no significance was found between the referrals in years of 2014–2016 and 2017–2019 (Table 3).

Table 4 presents unadjusted HR and adjusted HR from the regression analysis for WT2 for hip and knee replacement surgeries. The association between priority levels and WT2 was found to be mixed. For knee replacement, priority levels were not significantly related to WT2 (*p* = 0.3512) after adjusting for covariates of age group, diagnosis, patient’s preference, and year of referral. For hip replacement, the HR of having surgery was not proportional among priority groups (*p* = 0.0015). Patients with priority level 2 were less likely to have surgery sooner than those in priority level 3 with an adjusted HR of 0.447 (CI: 0.294–0.679) when decision for a surgery was make and the adjusted HR was 0.810 (CI: 0.162–0.824) at 182 days. No significant differences were found on hazard of having a surgery between patients in priority 1 and those in priority 3 after adjusting for other factors in the model for both of hip and knee replacements.

For WT2, the adjusted effects of age group, and year of referral were similar as those for WT1 while the effect of diagnosis type on WT2 was different and the effect of patient’s preference went to the opposite direction (Table 4). Patients for hip replacement with the osteoarthritis diagnosis were less likely to have surgery earlier than those of other diagnosis type (adjusted HR = 0.759, CI: 0.606–0.950) and diagnosis type was not significant among knee replacement patients (adjusted HR = 0.870, CI: 0.720–1.051). Patients choosing the next available surgeon indicated at referral were found to less likely to have surgery sooner than those preferring a specific surgeon in both of hip and knee replacements (adjusted HR = 0.798, CI: 0.683–0.932 for hip; HR = 0.863, 95% CI: 0.761–0.980 for knee) (Table 4).

Regarding TW from referral to surgery, Table 5 reports unadjusted HR and adjusted HR for hip and knee replacement surgeries. The effect of priority levels on TW was similar as those for WT1. The hazard of having a surgery for patients in priority levels 1 and 2 were greater than that of patients in priority 3. The adjusted HR for patients in priority level 1 were 2.999 (CI: 1.889–4.763) and 5.166 (CI: 3.364–7.934) for hip and knee replacements, respectively when the referral was made. The HR decreased to 2.112 (CI: 1.524–2.927) and 3.423 (CI: 2.566–4.565) for hip and knee replacement surgeries, respectively at 272 days after referral. The hazard of having a surgery for patients in priority 2 in comparison with those in priority 3 were 1.793 (CI: 1.179–2.728) for hip replacement and 3.221 (CI: 2.416–4.294) for knee replacement initially, and it became 1.615 (CI: 1.194–2.185) and 2.298 (CI: 1.860–2.839) at 272 days for hip and knee replacement, respectively. In contrast, patient’s preference indicated at referral was founded to be not significant for both hip and knee replacements (adjusted *p* = 0.4708 for hip and *p* = 0.2613 for knee), and referral form status was not significant as well. However, age group was significantly related to TW in that those under age 65 were less likely to have surgery earlier than those of age 65 or above (adjusted HR = 0.822, CI: 0.712–0.950 for hip; and HR = 0.819, CI: 0.726–0.923 for knee) after the referral was made. The effect of year of referral on TW was found to be similar as those for WT1.

### Post-hoc statistical power analysis

For the existing sizes of 808 and 1159 for hip and knee replacement surgeries, respectively, a minimum statistical power of 0.99 can be achieved for two-tailed tests at a family-wise type I error rate of 0.05 for eight multiple tests using the Bonferroni correction and adjusting for 5 covariates. The observed minimum HR of significance for priority level 1 and 2 vs level 3 was 0.477 (Tables 3 and 4). For each type of hip and knee surgeries, there were eight simultaneous tests for HRs for priority levels, four of which were for WT1. The proportions of patients in priority levels 1 and 2 vs level 3 for hip replacement were 0.72 and 0.88, respectively, and the proportions for knee replacement were 0.29 and 0.73. The extreme value of 0.88 produces the smallest p*(1-p) thus was used to determine the size that was the largest among those using the other proportions. To detect the minimum HR of 0.477 with statistical power of 0.99, the required approximate size was 422 based on the two-sided log-rank test at the family-wise type I error of 0.05 adjusting for five covariates in the model and for eight simultaneous tests using the Bonferroni correction.

## Discussion

A number of health regions in Canada shortened the WT1 after implementing a SEM [28–31, 51]. Our findings revealed that WT1 in patients with HKR was improved through the SEM at Eastern Health. A median WT1 pre-implementing OCI clinic was 168 days in 2006 to 210 days in 2010 [3–7]. After implementing the OCI clinic, a median WT1 was 49 days in priority 1, 75 days in priority 2, and 194.5 days in priority 3 for hip replacement surgery, and 54 days in priority 1, 82 days in priority 2, and 202 days in priority 3 for knee replacement surgery.

Studies have shown that incomplete initial referral form increased WT1 [52, 53]. Our study revealed that incomplete initial referral form was more likely to prolong WT1 than completed referral form. Choosing to see the next available surgeon for consultation may shorten WT1 [52, 54–56]. However, many patients are unlikely to consider switching surgeons [55]. Marshall et al. [54] found that patients were willing to wait a long time to meet an excellent reputation before accepting the next available surgeon. This study showed that most patients were willing to choose the next available surgeon and that choosing the next available surgeon reduced WT1. The relationship between age group and the likelihood of receiving a consultation booked early became nonsignificant while controlling independent variables. This is because those variables were probably positive confounders of the relationship between age group and WT1. Similarly, the effect of diagnosis on the probability of getting a consultation were nonsignificant when controlling possibly positive confounders.

Some previous studies have showed that WT2 was improved through the SEM [27–29, 31]. However, estimated Eastern Health median WT2 in this study were much higher than those in Canadian Institute for Health Information (CIHI) report that were from 97 days in 2014 to 149 days in 2019 for hip replacement, and from 108 days in 2014 to 157 days in 2019 for knee replacement [57]. This could because the median wait times from patients whose consultations or surgeries were delayed due to personal reasons were not included when determining the median wait times in the provincial wait times system [58]. The improvement of WT2 also depends on some factors such as the availability of non-physician operation room personnel or the availability of inpatient hospital beds for surgical patients may result in delaying elective surgeries [59, 60]. There was a nonsignificant difference in possibility of having a surgery between patients with priority 1 and those with priority 3 after controlling factors for HKR. This might be because urgent clinical ratings for HKR depend on clinical conditions such as pain, stiffness, function, and others decided by orthopedic surgeons [61, 62], which further studies need to investigate.

The main clinical indication for a total joint arthroplasty is osteoarthritis that accounted for over 90% of procedures for hip and knee [63]. The majority of HKR participants in the study were diagnosed with osteoarthritis. The study found that patients with hip osteoarthritis had to wait a longer time for their hip replacement surgery than those with other arthritis disorders such as rheumatoid arthritis. In contrast with rheumatoid arthritis that is best managed by a rheumatologist [63, 64], osteoarthritis is a progressive disease, and most patient with osteoarthritis will self-manage their disease such as changing their lifestyle, using over-the-counter analgesics, or looking for treatment by primary healthcare professional (e.g., physiotherapist, family doctors) before being referred to orthopedic surgeon for consultation. Of those requiring arthroplasty surgery, they will prepare themselves medically, socially, and functionally, as well as maintain their comorbidities under control before surgery [63]. These could result in wait longer times for surgery in patients with osteoarthritis than those with other arthritis disorders.

The study also revealed that choosing the next available surgeon for consultation did not improve WT2. This was a warning when surgeons were overburdened with large number of patients requiring surgery. Expanding the pool of participating surgeons for consultation is necessary to prevent the balance of waiting time across surgeon when the number of referrals choosing the next available surgeon increases. Developing a pool of surgeons for surgery should be also considered. The SEM in Eastern Health gave patients the choice of the next available surgeon for consultation, but surgeons still manage their own waiting list for surgery. In other words, when patients meet a surgeon for consultation, they have to wait for surgery performed by that surgeon, even if another surgeon is available. However, little is known if offering a choice of the next available for surgery can improve wait time for surgery, which need further studies.

An improvement of TW for HKR surgery through a SEM in Winnipeg Central Intake Service (WCIS) [27]. In contrast, the SEM at Eastern Health have not improved TW for HKR. The reports from the Fraser Institute [3–7] demonstrated that the median TW for orthopedic surgery in Newfoundland ranged from 257.6 days in 2006 to 436.8 days in 2010. Our findings were higher than values of the Fraser Institute’s reports. However, the use of values from the Fraser Institute’s reports should be used with cautions because the reports were based on surveys with small sample sizes from 14 to 19 questionnaires mailed out to the province [3–7]. Age group might impact patients willing for surgery. The risk of revision in patients under 65 is higher than in those at 65 or older [65–67], so this might make young patients are likely more unwilling to consider hip or knee replacement surgery than their counterparts [66–70]. After controlling factors, diagnosis was an individual factor influencing the probability of having a hip replacement surgery since patients were referred to by their physician, while the effect of diagnosis on the likelihood of getting a knee replacement surgery was nonsignificant. Therefore, the multiple Cox hazard regression model allows us to evaluate the effect of diagnosis on wait times for hip and knee replacement while simultaneously controlling negative confounding effects or positive confounding effects that reduce to underestimate or overestimate results, respectively.

### Strengths and limitations

This study provided an in-depth evaluation of the SEM and the priority classification to improve timely access to total joint arthroplasty in Newfoundland and Labrador’s Eastern Health region using Orthopedic Central Intake’s administrative data in Eastern Health. The administrative data allowed us to estimate WT1, WT2, and TW from referral to surgery with more accuracy than surveys. It is important to emphasize that wait times not only look at patients who have had surgery, but also all patients referred to the OCI. This study explored factors that significantly delayed having consultation or surgery in the SEM of the Eastern Health region from results of multivariable extended Cox proportional hazard regression models that allowed us to evaluate time-varying covariates.

Despite certain strengths, this study has certain limitations. We could not evaluate the improvement in WT1 pre-implementing OCI and post-implementing OCI based on administrative data because WT1 and TW data before implementing OCI clinic were not available. Therefore, we compared our findings with the Fraser Institute reports based on surveys with small sample sizes and low response rates, probably leading to bias. Information regarding gender, and comorbidities, and availability of resources is not available in the data sources in this study. The exclusion of large number of cases in the derivation of the sample may reduce the representativeness of the sample and bias results.

Given the study’s findings, qualitative research with all stakeholders, including health authorities, decision-makers, orthopedic surgeons, and family doctors should be required to elucidate relevant factors that matter and significantly impact wait times as well as decision-making for treatment based on patient’s perspectives and surgeon’s perspectives through the SEM. Moreover, the SEM should be governed at the provincial level, not only at regional levels in order to improve wait times management, including tracking, measuring, and monitoring across regions. This can allow patients to have equity in accessing orthopedic services regardless of health regions and facilitate better sharing resources across health regions.

## Conclusion

The study provided an insight into the improvement in wait time for consultation while using the priority classification in the single-entry model in Easter Health region of Newfoundland and Labrador. Hip and knee replacement patients with high priority were found to have consultation booked sooner than those with low priority, whereas the association between priority levels and wait time for surgery after decision for surgery was made has not well-established. Potential factors including initial referral form status and patient’s preference for a specific surgeon may delay consultation or influence patient’s willingness to have surgery through the SEM. Overall, it was found that the implementation of the single-entry model helped reduce total wait time and patient preference indicated at referral was not related to total wait time for surgery. Although age was not significantly related to wait time for consultation and for surgery after decision for surgery was make, patients under age 65 was found to wait longer for surgery after the referral made by a primary care physician than those aged 65 or above. Given the study’s findings and limitations, further studies could provide more insights about relevant factors that matter and significantly impact wait times.

## Data Availability

The data sets used and/or analysed during the current study are available from the corresponding author on reasonable request and upon approval of Easter Health, Newfoundland and Labrador (rpac@easternhealth.ca).

## Abbreviations

SEM: Single-Entry Model
OCI: Orthopedic Central Intake
WT1: Wait Time for Consultation
WT2: Wait Time for Surgery
OECD: Organization for Economic Cooperation and Development
HKR: Hip and Knee Replacement
COA: Canadian Orthopedic Association
MAWT: Maximum Acceptable Wait Time
TJAC: Total Joint Assessment Center
MCP: Medical Care Plan
HR: Hazard ratio
CI: Confidence interval
TC LHIN: Toronto Central Local Health Integration Network
CIHI: Canadian Institute for Health Information
